# Influence of ^68^Ga-DOTATOC on sparing of normal tissue for radiation therapy of skull base meningioma: differential impact of photon and proton radiotherapy

**DOI:** 10.1186/s13014-018-1008-z

**Published:** 2018-04-02

**Authors:** Falk Stade, Jan-Oliver Dittmar, Oliver Jäkel, Clemens Kratochwil, Uwe Haberkorn, Jürgen Debus, Stephanie E. Combs

**Affiliations:** 1 Department of Radiation Oncology, University Hospital of Heidelberg, Im Neuenheimer Feld 400, 69120 Heidelberg, Germany; 2 Deutsches Konsortium für Translationale Krebsforschung (dktk), Partner Site Heidelberg, Heidelberg, Germany; 3 Department of Radiation Oncology, Klinikum rechts der Isar, Technische Universität München, Ismaninger Straße 22, 81675 Munich, Germany; 4 Deutsches Konsortium für Translationale Krebsforschung (dktk), Partner Site Munich, Munich, Germany; 5 Department of Medical Physics in Radiation Oncology, German Cancer Research Center (DKFZ), Im Neuenheimer Feld 280, 69120 Heidelberg, Germany; 6 Heidelberg Ion Beam Therapy Center (HIT), Im Neuenheimer Feld 450, 69120 Heidelberg, Germany; 7 Department of Nuclear Medicine, University Hospital of Heidelberg, Im Neuenheimer Feld 400, 69120 Heidelberg, Germany; 8 Institute of Innovative Radiotherapy (iRT), Department of Radiation Sciences (DRS), Helmholtz Zentrum München, Ingolstädter Landstraße 1, 85764 Oberschleißheim, Germany

**Keywords:** ^68^Ga-DOTATOC-PET, Skull base meningioma, IMRT, Proton therapy, OAR

## Abstract

**Background:**

To evaluate the impact of ^68^Ga-DOTATOC-PET on treatment planning and sparing of normal tissue in the treatment of skull base meningioma with advanced photons and protons.

**Methods:**

From the institutional database consisting of 507 skull base meningiomas 10 patients were chosen randomly for the present analysis. Target volume definition was performed based on CT and MRI only, as well as with additional ^68^Ga-DOTATOC-PET. Treatment plans were performed for Intensity Modulated Radiotherapy (IMRT) and proton therapy using active raster scanning on both target volumes. We calculated doses to relevant organs at risk (OAR), conformity indices as well as differences in normal tissue sparing between both radiation modalities based on CT/MRI planning as well as CT/MRI/PET planning.

**Results:**

For photon treatment plans, PET-based treatment plans showed a reduction of brain stem D_max_ and D_median_ for different levels of total dose. At the optic chiasm, use of ^68^Ga-DOTATOC significantly reduces D_max_; moreover, the D_median_ is reduced in most cases, too. For both right and left optic nerve, reduction of dose by addition of ^68^Ga-DOTATOC-PET is minimal and depends on the anatomical location of the meningioma. In protons, the impact of ^68^Ga-DOTATOC-PET is minimal compared to photons.

**Conclusion:**

Addition of ^68^Ga-DOTATOC-PET information into treatment planning for skull base meningiomas has a significant impact on target volumes. In most cases, PET-planning leads to significant reductions of the treatment volumes. Subsequently, reduced doses are applied to OAR. Using protons, the benefit of additional PET is smaller since target coverage is more conformal and dose to OAR is already reduced compared to photons. Therefore, PET-imaging has the greatest margin of benefit in advanced photon techniques, and combination of PET-planning and high-precision treatment leads to comparable treatment plans as with protons.

## Background

Radiation therapy (RT) is a central treatment alternative in patients with skull base meningiomas [1]. Since neurosurgical resection can be associated with significant morbidity due to the complex anatomical structures of the skull base, especially if complete resections are anticipated, RT is generally associated with very low toxicity and local control rates are above 80–90% even after 10 or 20 years [2, 3]. However, the intricate anatomy of the skull base also poses a challenge to the radiation oncologist: The goal is the delivery of necessary doses to the target volumes, while keeping the dose outside the target volumes, especially to Organs at Risk (OAR) as low as possible [4, 5]. Therefore, advanced RT techniques such as stereotactic radiotherapy, intensity modulated radiotherapy (IMRT) or particle therapy are recommended for skull base lesions.

The standard imaging protocols for treatment planning of skull base meningiomas include contrast-enhanced CT and MRI. Previously, it has been shown that addition of ^68^Ga-DOTATOC-PET can improve target volume definition [6–13]. Compared to CT or MRI, ^68^Ga-DOTATOC PET/CT demonstrated an improved sensitivity in meningioma detection when compared to contrast-enhanced MRI. Especially skull base lesions or meningiomas obscured by imaging artifacts or calcifications can be detected more precisely with additional PET; in cases with uncertain or equivocal results on MRI ^68^Ga-DOTATOC-PET-Imaging can help confirm the diagnosis of meningioma [14]. For meningiomas with extension into soft tissues, especially after surgical interventions, PET-planning significantly reduces treatment volumes; for bony meningiomas, PET-planning generally enhances detection of the bony lesions and often leads to a significant enlargement of volumes [10]. Some authors, such as Graf et al. reported that target volumes can be reduced overall by about 10% [8, 11]. Additional precision of added ^68^Ga-DOTATOC-PET has been reported by using PET-MRI combination devices minimizing any positioning or matching errors [9, 12, 14].

Thus, the contribution to target volume reduction has been shown. However, it is unclear whether this reduction, does actually result in a meaningful and clinically reduction of dose to OAR. Moreover, it is unclear of this modification of target volumes is independent of the radiation technique applied.

Therefore, in the present work we determined the impact of target volume modification by use of ^68^Ga-DOTATOC-PET for RT planning in patients with skull base meningiomas. We calculated the potential of dose reduction for different high-precision techniques comparing advanced photons to protons.

## Methods

### Patient characteristics

From the institutional database a group of 10 patients with skull base meningiomas treated with RT was chosen randomly from a group of 507 patients treated with high-precision RT [3]. Nine patients were female, one patient was male. The median age was 58 years (range 42–70 years). In 8 out of 10 patients histologically confirmed diagnosis of WHO Grade I meningioma was present, in two patients diagnosis of low-grade meningioma was imaging based. The median planning tumor volume (PTV) was 50 cm^3^ (range 19.2 cm^3^–218.4cm^3^). All patients had been treated with RT as described previously [10, 15]. For treatment planning all patients had been positioned by an individual mask fixation either made of Scotch Cast™ or mask systems made of thermoplastic material as described previously. All patients had received contrast enhanced CT and MRI, as well as ^68^Ga-DOTATOC-PET imaging for RT treatment planning. The study was approved by the Ethic’s Committee of the Medical Faculty, University of Heidelberg.

### Target volume

**Fig. 1:**
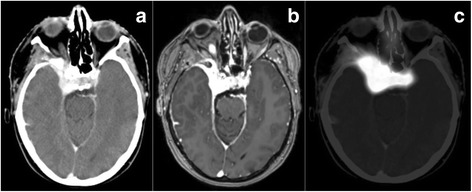
Imaging of a typical skull base meningioma on **a** contrast-enhanced CT, **b** contrast-enhanced MRI and **c** ^68^Ga-DOTATOC-PET

Target volumes were re-evaluated from all 10 patients and two sets of gross tumor volumes (GTV) were defined. CT and MRI at 1-3 mm slice slickness were used for treatment planning and therefore for the present analysis. After initial automatic and additional manually fine-tuned image fusion of CT, MRI and ^68^Ga-DOTATOC-PET for each patient, target volumes were drawn manually from experienced radiation oncologists with expertise in the field of radiation oncology and nuclear medicine. One volume was based on contrast-enhanced CT and MRI imaging only; the second volume additionally included ^68^Ga-DOTATOC-PET information. Figure 1 shows all three imaging modalities in a typical patient with a skull base meningioma. We followed our imaging protocols as published previously [6, 10, 14, 16]. We defined the meningioma-SUV for each patient: For that, typical meningioma tissue on MRI/CT was identified on the PET- image and the tracer uptake for that region was documented. Then, we calculated the SUV_max_ for meningioma tissue in relation to the tracer-uptake in normal tissue. By this procedure we defined a specific individual meningioma-SUV for each patient by referencing the SUV_max_ to a region of typical meningioma tissue visible in CT and MR. The median patients-specific value was 58% (range 54% – 62%). Additionally a clinical target volume (CTV) was determined adding 1 mm safety margin, as well as a planning target volume as described previously [3]. The PTV was added based on institutional standards.

### Treatment planning

For all patients, we calculated treatment plans for intensity modulated radiotherapy (IMRT) as well as proton therapy using the active raster scanning method. For IMRT planning the Oncentra Masterplan (Nucletron, Elekta, Stockholm, Sweden) software was used as described previously [3]. A template of 9 beam angles at 40 ° distances was used and modified as needed for optimal target volume coverage and OAR sparing. For proton therapy, the *syngo* RT Planning System (Siemens, Erlangen, Germany) was used. All plans were calculated using horizontal proton beams. The use of horizontal proton beams was generally used at the time of the analysis to treat most skull base lesions at the Heidelberg Ion beam Therapy Center and thus used for this analysis, which was performed also for internal treatment optimization [15].

All plans were optimized to a target dose of 54 Gy in 1.8 Gy single fractions as well as plans for 57.6 Gy in 1.8 Gy single fractions based on the two main published dosing concepts for skull base meningiomas [2, 3]. The aim was to cover 95% of the treatment volume with at least 90% of the median prescribed dose following ICRU (international commission on radiation units & measurements) guidelines for treatment planning and reporting.

**Fig. 2:**
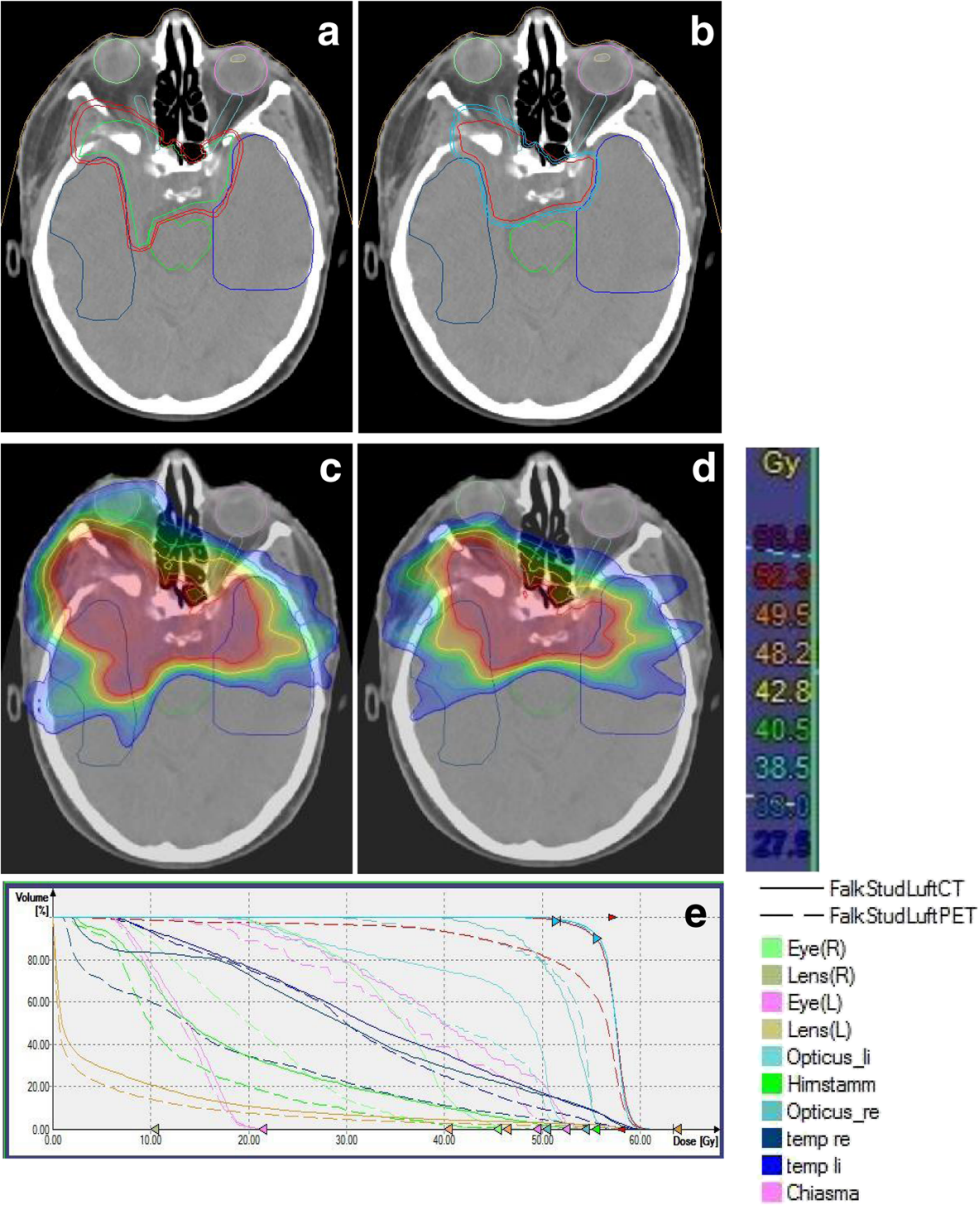
Target volume based on CT and MRI **a** and target volume based on CT, MRI as well as ^68^Ga-DOTATOC-PET **b**. Images **c** and **d** show the corresponding treatment plans with photon IMRT. Image **e** shows the Dose-Volume-Histogram comparing dose to the target and to OAR

For both modalities, treatment plan optimization and OAR sparing followed the QUANTEC (Quantitative Analyses of Normal Tissue Effects in the Clinic) recommendations to remain below a maximal toxicity rate of 5% at 5 years. For example, for brain stem constraints a maximal dose of 59 Gy at 10 cm^3^ and/or 54 Gy to the whole brain stem volume was allowed [17]. The maximal dose to the optic nerves and chiasm was set at 55 Gy [18]. Highest priority was dose to the brain stem, followed by the optic system. Treatment planning was optimized multiparametrically until the best compromise between target volume coverage and OAR sparing was achieved. All plans were reviewed and accepted by a team of experienced radiation oncologists. (12). Figure 2 depicts the differences in target volumes and corresponding IMRT treatment plans of a typical skull base meningioma case.

### Statistical analysis

To assess doses to OAR, we calculated D_max_ and D_median_ of brain stem, chiasm and left and right optic nerves.

Descriptive statistical analysis was performed using SPSS-IBM Version 21. Differences were described using the Wilcoxon-text for paired samples and the *p*-value was generated for median and maximal doses to each OAR.

## Results

### Skull base meningioma treatment plans with IMRT: Dose to OAR

For plans calculated for a total dose of 54 Gy, in 9 out of 10 patients PET-based target volumes lead to a reduction of brain stem D_max_ which was highly significant at *p* = 0.0097. In 8 out of 10 patients, a reduction of the brain stem D_median_ was observed which was also significant at *p* = 0.037. Results are shown in Table 1.

**Table 1:** Dose to the organs at risk (OAR) in IMRT treatment plans (54 Gy total dose)

Parameter	CT-MRI (Gy, median, range)	CT-MRI-PET (Gy, median, range)	*p*-value
*Brain Stem*			
D_max_	52.4 (39.8–55.3)	51.4 (39.7–54.7)	0.007*
D_median_	11.9 (2.8–22.4)	5.5 (3.7–16.1)	0.037*
*Optic Chiasm*			
D_max_	52.5(5.7–55.2)	51.0 (5.0–53.5)	0.007*
D_median_	39.9 (2.8–53.5)	37.9 (2.4–49.9)	0.203
*Left optic nerve*			
D_max_	53.8 (3.1–56.1)	52.3 (3.1–54.6)	0.017*
D_median_	40.9 (1.5–52.6)	37.1 (1.5–52.9)	0.063
*Right optic nerve*			
D_max_	49.5 (1.5–55-7)	48.4 (1.5–55.0)	0.022*
D_median_	28.2 (0.8–54.0)	24.7 (0.8–53.2)	0.047*

*Significant difference <0.05

Table 2 shows dose calculations for plans with 57.6 Gy IMRT; total dose reduction of D_max_ could be achieved in 8 out of 10 patients (*p* = 0.013); for D_median_ the IMRT plans of 6 out of 10 patients showed a reduced dose (*p* = 0.241).

**Table 2:** Dose to the organs at risk (OAR) in IMRT treatment plans (57.6 Gy total dose)

Parameter	CT-MRI (Gy, median, range)	CT-MRI-PET (Gy, median, range)	*p*-value
*Brain Stem*			
D_max_	55.1 (47.0–58.1)	54.7 (43.2–58.72	0.013*
D_median_	13.4 (3.0–21.3)	10.33 (2.8–22.4	0.241
*Optic Chiasm*			
D_max_	53.1(7.3–57.9)	51.8 (4.2–57.5)	0.032*
D_median_	42.6 (3.36–51.0)	41.0 (2.8–53.5)	0.007*
*Left optic nerve*			
D_max_	54.5 (5.9–58.7)	53.6 (2.3–58.6)	0.036*
D_median_	42.4 (2.2–55.6)	35.3 (1.5–55.7)	0.017*
*Right optic nerve*			
D_max_	52.1 (1.8–56.7)	51.2 (1.6–57.3)	0.037*
D_median_	33.7 (0.9–54.5)	28.1 (0.9–54.2)	0.013*

*Significant difference <0.05

For the optic chiasm, in treatment plans calculated for total dose of 54 Gy, 9 out of 10 patients had a reduction of D_max_ to the optic chiasm when treatment planning included 68Ga-DOTATOC-PET (*p* = 0.007). Moreover, D_median_ was reduced in seven out of 10 patients however not statistically significant (*p* = 0.23). When the total dose was increased to 57.6 Gy, 8 out of 10 patients had a reduced D_max_ to the optic chiasm based on PET-planning (*p* = 0.032) and the D_median_ was reduced in 9 out of 10 patients (p = 0.007) which was significant in both cases.

For both right and left optic nerve, reduction of dose by addition of ^68^Ga-DOTATOC-PET is minimal compared to the impact observed from the brain stem and optic chiasm. However, the impact of PET depends clearly on the anatomical location of the meningioma. For large skull base lesions, one (or both) optic nerves are often within or very close to the target volume, thus the impact observed is only minimal. However, for both optic nerves, dose reduction is significant with addition of PET, for 54 Gy plans as well as for 57.6 Gy plans. When comparing both dosing regimens the margin of benefit is larger in the 57.6 Gy dataset. Data are shown in Table 1 for 54 Gy plans, and in Table 2 for 57.6 Gy plans.

### Skull base meningioma treatment plans with Protons: Dose to OAR

Tables 3 and 4 summarize the data for D_max_ and D_median_in proton treatment plans. Both for 54 Gy and for 57.6 Gy the impact of target volume modification by ^68^Ga-DOTATOC PET information is minimal compared to IMRT plans. Generally, compared to the IMRT values, it can be seen that the dose reduction is less with protons than with IMRT. Generally, only the median dose was significantly lower with PET-planning. This was true for all OAR evaluated.

**Table 3:** Dose to the organs at risk (OAR) in proton treatment plans (54 Gy total dose)

Parameter	CT-MRI (Gy, median, range)	CT-MRI-PET (Gy, median, range)	*p*-value
*Brain Stem*			
D_max_	52.8 (51.2–53.2)	52.8 (51.9–54.6)	0.86
D_median_	11.7 (0.7–40.1)	5.7 (0.4–35.3)	0.008*
*Optic Chiasm*			
D_max_	52.6 (1.8–53.4)	52.8 (1.81–54.7)	0.767
D_median_	39.3 (0.3–53.4)	39.7 (0.3–49.4)	0.038
*Left optic nerve*			
D_max_	52.8 (0.4–56.4)	52.7 (0.4–55.7)	0.678
D_median_	38.2 (0–51.9)	29.9 (0–51.6)	0.008*
*Right optic nerve*			
D_max_	51.6 (0.1–53.0)	51.9 (0.1–54.3)	0.674
D_median_	16.2 (0–51.2)	6.4 (0–51.2)	0.036*

*Significant difference <0.05

**Table 4:** Dose to the organs at risk (OAR) in proton treatment plans (57.6 Gy total dose)

Parameter	CT-MRI (Gy, median, range)	CT-MRI-PET (Gy, median, range)	*p*-value
*Brain Stem*			
D_max_	54.3 (52.3–56.0)	54.6 (52.4–57.6)	0.859
D_median_	12.5 (0.7–41.5)	5.7 (0.48–35.8)	0.008*
*Optic Chiasm*			
D_max_	53.4 (1.9–55.8)	53.6 (1.9–55.3)	0.767
D_median_	42.0 (0.3–51.3)	40.4 (0.3–50.3)	0.139
*Left optic nerve*			
D_max_	53.7 (0.4–57.2)	53.2 (0.4–57.5)	0.953
D_median_	38.6 (0.0–52.3)	29.9 (0.0–51.9)	0.008
*Right optic nerve*			
D_max_	52.9 (0.2–53.8)	52.7 (0.2–55.2)	0.953
D_median_	16.3 (0.0–51.3)	7.0 (0.0–51.5)	0.021*

*Significant difference <0.05

## Discussion

By adding ^68^Ga-DOTATOC-PET to target volume definition in radiation oncology significant reduction of target volumes compared to CT and MRI only can be achieved. The present manuscript describes the potential to reduce dose to OAR by adding PET to treatment planning. Modification of target volumes reduces dose to OAR with photon radiotherapy. The effect is highest when OAR are not included in the target volumes. For the skull base tumors evaluated, the greatest benefit is seen for the brain stem as well as the optic chiasm. For patients treated with protons, the PET-effect is minimal, mainly because dose conformality and dose outside the target is already reduced due to the physical properties of particle beams.

To optimize the therapeutic window in radiation oncology minimizing dose to normal tissue is an essential aim. Modern RT techniques, such as stereotactic treatment, IMRT or particle therapy continuously led to increased dose conformality to the target together with reduction of dose to normal tissue; the step from advanced photons to particle therapy, e.g. protons, is characterized especially by the reduction of the integral dose.

^68^Ga-DOTATOC-PET has been established for diagnosis and treatment planning of meningioma. Afshar-Oromieh et al. have shown in 134 patients investigated by both modalities that 190 meningiomas were detected by ^68^Ga-DOTATOC PET/CT and only 171 by contrast-enhanced MRI; moreover, they could show that adding the knowledge from PET-imaging 4 out of 19 meningiomas were only detectable on MRI knowing the additional information from the PET-imaging; this lead to an overall detection rate of 92% [6]. For treatment planning addition of ^68^Ga-DOTATOC information modified target volumes significantly: Mostly lesions extending into soft tissue e.g. parapharyngeal meningiomas, seem larger on MRI than the real volume as shown on PET; for bony meningiomas, which are often difficult to identify by MRI and often only visibly on CT imaging in bone windows target volumes are enlarged with PET-target volume definition. However, to date, no prospective trials comparing target volume definition based on MRI versus PET have been performed, and most data on radiotherapy for meningiomas derives from MRI-based treatment planning. Therefore, one must keep in mind that modifications in target volumes might potentially convey to changes in clinical outcome.

The potential of PET-planning to reduce treatment volumes and thus potentially increase dose to the target while reducing dose to OAR has been evaluated only in few trials. One planning study evaluating FDG-PET/CT during radiotherapy in patients with esophageal cancer showed a decreased target volume by addition of PET-CT during RT. Moreover, the planning study suggested that due to smaller volumes and reduced dose to OAR doses of up to 66 Gy can be applied safely [19].

However, in contrast to low-grade meningiomas of the skull base, dose escalation plays a role in lung cancer. For meningiomas, high local control rates can most likely not be increased by addition of dose, and in the past, several studies have shown that even slightly higher doses (52.2 Gy compared to 57.6 Gy) do not convey into higher local control rates. Thus, the rationale for dose sparing to OAR is more likely in terms of long-term risk reduction, e.g. secondary malignancies or functional changes. The idea of integral dose reduction is strongly associated with proton therapy [20]. Due to the physical properties of protons sparing of normal tissue outside the target volumes is possible. Several groups have calculated an alleged risk reduction for secondary cancers, neurocognitive decline or other side effects [21–25]. However, to date, no prospective studies have confirmed this clinical hypothesis.

## Conclusions

In conclusion, the potential to further reduce dose to OAR with protons is minimal. However, with IMRT, the benefit is significant for all OAR evaluated. Therefore, with advanced treatment planning improvement of high-end IMRT is achieved moving treatment plans closer to those achieved by particle therapy. Therefore, comparing of advanced photons with high-end imaging for treatment planning to proton therapy potentially leads to comparable results, in terms of tumor control rates and side effects. However, this must be confirmed in prospective clinical trials, of which some are already underway.

## Abbreviations

CI: Conformity index
GTV: Gross tumor volume
HI: Homogeneity index
ICRU: International commission on radiation units & measurements
IMRT: Intensity Modulated Radiotherapy
OAR: Organs at risk
PTV: Planning tumor volume
QUANTEC: Quantitative Analyses of Normal Tissue Effects in the Clinic
RT: Radiation therapy
SUV: Standard-uptake-value
